# Mammographic sensitivity as a function of tumor size: A novel estimation based on population-based screening data

**DOI:** 10.1016/j.breast.2020.12.003

**Published:** 2020-12-09

**Authors:** Jing Wang, Pam Gottschal, Lilu Ding, DaniëlleW.A van Veldhuizen, Wenli Lu, Nehmat Houssami, Marcel J.W. Greuter, Geertruida H. de Bock

**Affiliations:** a University of Groningen, University Medical Center Groningen, Department of Epidemiology, Groningen, the Netherlands; b Department of Epidemiology and Health Statistics, School of Public Health, Tianjin Medical University, Tianjin, China; c Sydney School of Public Health, Faculty of Medicine and Health, University of Sydney, Australia; d University of Groningen, University Medical Center Groningen, Department of Radiology, Groningen, the Netherlands; e Robotics and Mechatronics (RaM) Group, Faculty of Electrical Engineering Mathematics and Computer Science, Technical Medical Centre, University of Twente, Enschede, the Netherlands

**Keywords:** Breast Neoplasms, Mass screening, Mammography, Sensitivity, Tumor growth, FN, false negative, TP, true positive, TVDT, tumor volume doubling time

## Abstract

**Background:**

Instead of a single value for mammographic sensitivity, a sensitivity function based on tumor size more realistically reflects mammography’s detection capability. Because previous models may have overestimated size-specific sensitivity, we aimed to provide a novel approach to improve sensitivity estimation as a function of tumor size.

**Methods:**

Using aggregated data on interval and screen-detected cancers, observed tumor sizes were back-calculated to the time of screening using an exponential tumor growth model and a follow-up time of 4 years. From the observed number of detected cancers and an estimation of the number of false-negative cancers, a model for the sensitivity as a function of tumor size was determined. A univariate sensitivity analysis was conducted by varying follow-up time and tumor volume doubling time (TVDT). A systematic review was conducted for external validation of the sensitivity model.

**Results:**

Aggregated data of 22,915 screen-detected and 10,670 interval breast cancers from the Dutch screening program were used. The model showed that sensitivity increased from 0 to 85% for tumor sizes from 2 to 20 mm. When TVDT was set at the upper and lower limits of the confidence interval, sensitivity for a 20-mm tumor was 74% and 93%, respectively. The estimated sensitivity gave comparable estimates to those from two of three studies identified by our systematic review.

**Conclusion:**

Derived from aggregated breast screening outcomes data, our model’s estimation of sensitivity as a function of tumor size may provide a better representation of data observed in screening programs than other models.

## Highlights

• Mammographic sensitivity is a key indicator of screening effectiveness.
• Previous model using logistic function might overestimate size-specific sensitivity.
• Our model showed that sensitivity increased from 0 to 85% for tumor sizes from 2 to 20 mm.
• Our model may provide a better representation of data observed in screening programs.

## 1 Introduction

Breast cancer is the most common cancer and one of the main causes of death in European women, approximately one in seven women will develop breast cancer by the age of 75 [1]. In recent decennia, mammography screening has been introduced in many countries. Studies have shown that screening can detect breast cancer at an earlier stage which will reduce treatment burden and improve survival [[2], [3], [4]]. However, there are ongoing debates on whether screening does more harm than good and on the related optimization of screening strategy. To inform these debates, it is important to evaluate breast cancer screening programs considering indicators of both long-term, such as decreasing burden of breast cancer-specific treatment and mortality benefit, and short-term indicators such as mammography sensitivity and specificity [5,6]. In this contribution, we focus on the estimation of mammographic sensitivity as a function of tumor size, which is highly relevant for the evaluation of screening programs [7,8]. However, we cannot measure sensitivity directly as there are no methods to determine the amount of asymptomatic cancers that are detectable by screening [9].

Whereas most studies give one constant estimate for the sensitivity of mammography, Weedon-Fekjær et al. developed a logistic model to estimate the sensitivity of mammography as a function of tumor size [10,11]. In their studies, the sensitivity was estimated simultaneously with a continuous growth model utilizing breast cancer screening data, and back-calculation methods were used to estimate tumor size at screening from tumor size distributions of clinically detected tumors. Inspired by this approach, Swedish researchers estimated the sensitivity not only based on tumor size, but also breast density [12,13]. What is remarkable about the findings of their studies is that the sensitivity is 100% for tumors varying in size from 15 to 20 mm and over. However, this seems unlikely, as several studies showed that approximately 10–30% of all screen-detected tumors are larger than 20 mm, which indicated that at least a part of these tumors are missed at the size of 15–20 mm [[14], [15], [16]]. In addition, studies have shown that even tumors larger than 50 mm can be invisible on mammography [17,18].

In this study, we therefore aimed to provide a novel method to improve the estimates of mammography sensitivity as a function of tumor size by using aggregated data reported from a national population breast cancer screening program. We anticipate that the sensitivity function can be integrated into modeling studies focusing on the evaluation of breast cancer screening programs, which in turn can provide valuable evidence for the optimization of screening strategies.

## 2 Methods

A sensitivity model estimating mammographic sensitivity as a function of tumor size was developed in this study. To develop this sensitivity model, empiric data on number and sizes of screen-detected and interval cancers from a population-based breast screening program and back-calculation of these tumor sizes to the screening moment were used to determine the number of false negatives (FN). The model was externally validated on published data identified by a systematic review.

### 2.1 The sensitivity model: a description

In our sensitivity model, the probability of finding a tumor with volume V at screening moment i is based on the well-known formula for sensitivity:

$$S_{i}\left(V\right)=\frac{TP_{i}\left(V\right)}{TP_{i}\left(V\right)+FN_{i}\left(V\right)}$$

where $S_{i}\left(V\right)$ is the sensitivity to detect a tumor of volume *V* at screening round i, and $TP_{i}\left(V\right)$ and $FN_{i}\left(V\right)$ are the number of true-positives and false-negatives at screening round i as a function of tumor volume V respectively.

**Fig. 1:**
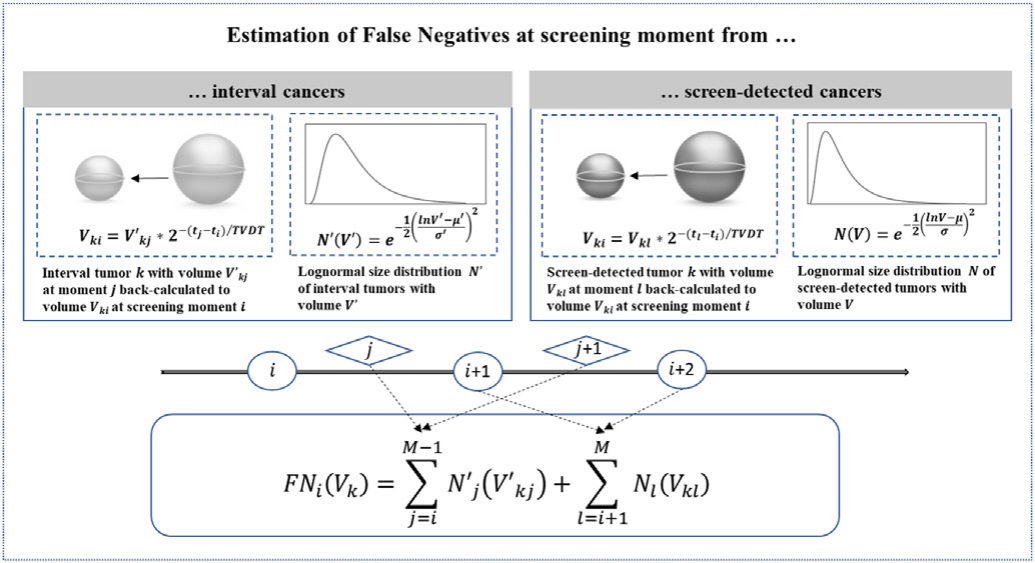
Estimation of false-negatives (FNs), where $V_{ki}$ represents the volume of a tumor *k* at $t_{i}$, $V_{kj}^{\prime }$ and $V_{kl}$ represent the volume of a tumor *k* during screening intervals (j,j+1,…) and at subsequent screening rounds (i+1,i+2,…) respectively, and the corresponding numbers of tumors are represented as $N_{j}^{\prime }$and $N_{l}$. TVDT = Tumor volume doubling time.

To determine the number of false-negatives as a function of volume we use the assumption that the undetected tumors at screening round i grow larger over time and will eventually be detected either at a subsequent screening round or as an interval cancer (Fig. 1).

Let the number of screen-detected tumors at screening moment $t_{i}$ be equal to $N_{i}$, where i = 1,2, …M runs over the total number M of screening moments in the screening program. Let the size of a tumor k which is screen-detected at screening moment $t_{i}$ be equal to $V_{ki}$. Let the number of interval tumors between screening moment i and i+1 be equal to $N_{j}^{\prime }$, where j = 1…*M*-1- with corresponding tumor sizes $V_{kj}^{\prime }$. Assume for each tumor k an exponential growth model where the volume at screening moment i is given by: $V_{ki}=V_{k}^{0}*2^{\left(t_{i}-t_{0}\right)/TVDT}$, where $V_{k}^{0}$ is the starting volume at time t_0_ and TVDT is the tumor volume doubling time. Now, from the tumor size detected by screening or intervals later than screening moment i, we can calculate back the tumor size at the time of screening using the exponential growth model.

If we assume an interval tumor is found at time $t_{j}$, then the size of this tumor at screening moment $t_{i}$ will be equal to: $V_{ki}=V_{kj}^{\prime }*2^{-\left(t_{j}-t_{i}\right)/TVDT}$. Also, the size of a tumor found in one of the subsequent screening rounds l >i can be calculated back in time to the size at the time of screening. If we assume a screen-detected tumor is found at time $t_{l}$ then the size at screening moment $t_{i}$ will be equal to:$\;V_{ki}=V_{kl}*2^{-\left(t_{l}-t_{i}\right)/TVDT}$. Now, we can estimate the number of false negatives $FN_{i}\left(V\right)$ with volume V at screening moment i by

$$FN_{i}\left(V\right)=\overset{M-1}{\underset{j=i}{\sum }}N_{j}^{\prime }\left(V_{kj}^{\prime }\right)+\overset{M}{\underset{l=i+1}{\sum }}N_{l}\left(V_{kl}\right)$$

i.e. the number of back-calculated interval tumors ( $N_{j}^{\prime }$) with size V at the time of screening plus the number of back-calculated subsequent screen-detected tumors ( $N_{l}$) with size V at the time of screening $t_{i}$. Together with the number of detected tumors at screening momenti given by $TP_{i}\left(V\right)$ = $N_{i}\left(V\right)$, we can calculate the sensitivity $S_{i}\left(V\right)$ as a function of volume V.

### 2.2 The sensitivity model: input parameters

#### 2.3 Tumor growth

For tumor growth, tumors were assumed to be spherical and to grow exponentially with a constant volume doubling time [19]. In this study, the tumor volume doubling time for women aged 50–70 years old was on average 157 days [20]. For the distribution of the screen-detected and interval tumor sizes, we used data from the Dutch breast cancer screening program from 2004 to 2009 [14, Table 1]. The data from the first screening round was excluded as it is well known that in the first screening round relatively more and larger tumors are found compared to the subsequent screening rounds [21]. We used a nonlinear least-squares method to obtain the parameters of the log-normal tumor size distributions of the screen-detected and interval cancers found in the screening.

**Table 1:** Tumor size distribution of screen-detected cancers and interval cancers^a^.

T categories (tumor size)	Distribution of screen-detected cancers	Distribution of interval cancers
T1a (≤5 mm)	7.7%	2.2%
T1b (>5 mm and ≤10 mm)	24.5%	8.9%
T1c (>10 mm and ≤20 mm)	49.0%	40.0%
T2 (>20 mm and ≤50 mm)	17.9%	41.1%
T3 (>50 mm)	0.9%	7.8%

a Data source: National evaluation of breast cancer screening in the Netherlands, 1990–2011/2012.

#### 2.4 Time since previous screening

We assumed biennial screening frequency as used in the Dutch screening program and many population-based breast screening programs. The maximum delay time in diagnosis after a false negative breast assessment in recalled women in a biennial screening program was 1251 days, which was rounded up to four years [22]. A median time from biennial screening to diagnosis of interval cancers of 502 days was used [23]. The time between the diagnosis of an interval cancer which had a possible false-negative result in the previous one or two screenings rounds was therefor set at 502 and 1232 (two years plus 502 days) days respectively. The time between the diagnosis of a screen-detected cancer which had a possible false-negative result in the previous one or two screening rounds was set at two years and four years, respectively.

#### 2.5 Analysis of the results of the sensitivity model

The main outcome, i.e. tumor size-specific sensitivity estimated from the developed model was described graphically. To evaluate the uncertainty of our model, univariate sensitivity analyses were performed by varying input values of model parameters. Lastly, external validation of the developed model was conducted based on published data identified by a systematic review.

#### 2.6 Analysis on the assumptions of the sensitivity model

We performed sensitivity analyses to evaluate the uncertainty of our model. The tumor volume doubling time was set to the lower and upper bounds of the 95% confidence interval (CI), which were 121 days and 204 days respectively [20], and the follow-up time between a screen-detected or interval cancer and the previous screening rounds was set at 2 and 6 years.

#### 2.7 External validation of the sensitivity model

For external validation, we performed a systematic search in PubMed to find related articles focusing on mammography sensitivity and tumor size. The keywords used in the search included “breast carcinoma”, “mammography”, “sensitivity and specificity”, and “tumor size”. If the study reported observed sensitivities and related tumor size from a population-based screening program, then it would be included for further comparison. To ensure recent mammographic methods were used, the searches focused on relevant articles published from January 1, 2000 to August 1, 2020. Two authors searched the literature independently. A detailed description of the search strategies can be found in Supplementary data. From the included studies, the reported sensitivity was compared to our model.

## 3 Results

### 3.1 Mammography sensitivity according to the model

**Fig. 2:**
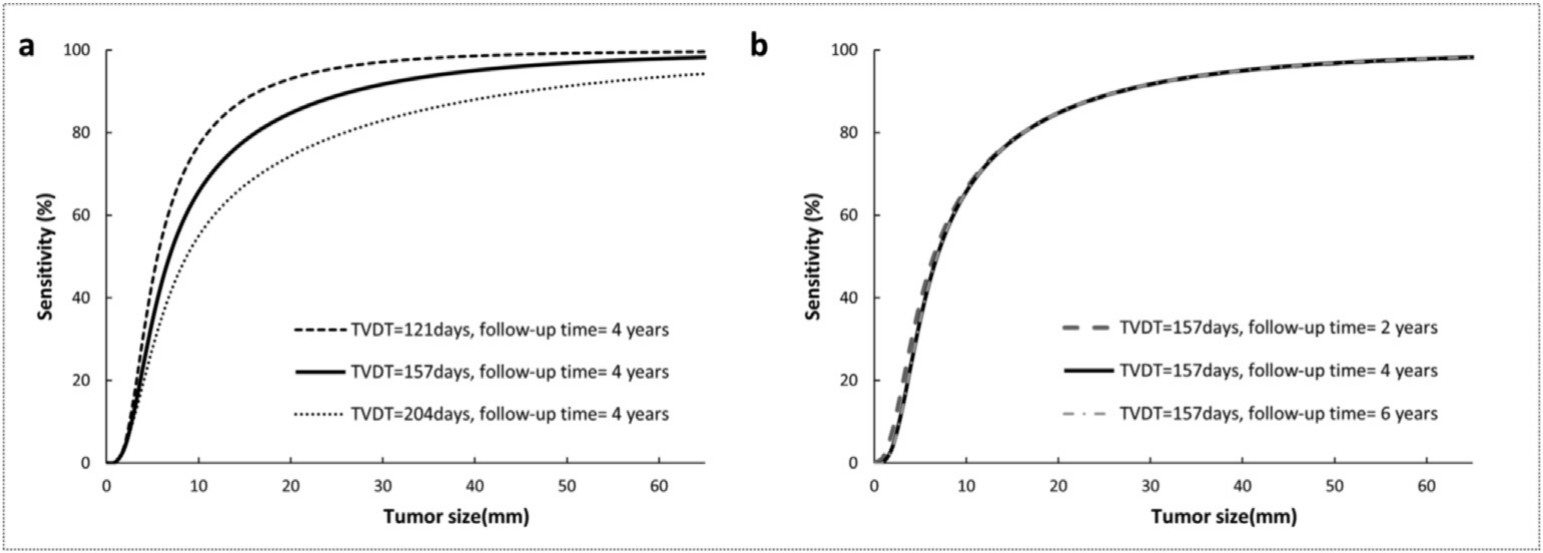
The estimated mammographic sensitivity as a function of tumor size. Solid line in a and b: the sensitivity model using a TVDT of 157 days and a follow-up time of 4 years; a: the sensitivity analysis of varying TVDT (121 and 204 days), and b: the sensitivity analysis of varying follow-up time (2, and 6 years). TVDT = tumor volume doubling time.

The aggregated data of 22,915 screen-detected cancers and 10,670 interval cancers were used for the estimation of tumor size distributions. For screen-detected cancers, the mean diameter and corresponding standard deviation (mm) were 14.0 (95%CI: 10.6–18.4) and 1.93 (95%CI: 1.52–2.46), while for interval cancers, these were 20.9 (95%CI: 18.5–23.8) and 1.77 (95%CI: 1.58–1.95), respectively. Given a TVDT of 157 days and a 4-year follow-up, the model showed a sensitivity function which continuously increased from 0 to 85% for tumor diameters between 2 and 20 mm (Fig. 2: Solid line). The estimated sensitivity at 5, 10, 15 and 50 mm was 35%, 65%, 78%, and 97%, respectively.

### 3.2 Analysis on the assumptions

The mammography sensitivity increased with a decrease in TVDT (Fig. 2). When the TVDT was set at the upper and lower limits of its confidence interval, the sensitivity for a 20-mm tumor became 74% and 93%, respectively. Unlike TVDT, different follow-up times only had a minor impact on our sensitivity model. Increasing the follow-up time to 6 years did not affect the sensitivity. With a shorter follow-up time (2 years), the sensitivity as found with our sensitivity model slightly increased when tumor size was smaller than 10 mm. Specifically, the sensitivity was 39% and 67% for a 5 and a 10 mm tumor, respectively, whereas for larger tumor sizes, the sensitivity remained nearly unchanged.

### 3.3 External validation of the sensitivity model

After literature searching and screening, three studies were included [[24], [25], [26]]. All three studies reported mammography sensitivity and its related mean tumor size. To allow comparison, our estimated sensitivity at the mean tumor size reported in the literature (Table 2) was used. Specifically, our model gave reliable estimations which were comparable to two of the included studies [25,26]. However, the sensitivity was slightly underestimated compared to that of Cawson et al. [24].

**Table 2:** Validation results based on screening data.

Reference	Mean tumor size (mm)	Observed sensitivity (%)	Estimated sensitivity at the same size (%)
Cawson et al. [24]	18.7	90.4 (84.7–94.6)	83.6
Moshina et al. [25]	15.6	77.6 (75.6–79.6)	79.2
Skaane et al. [26]	13.6	76.2 (72.2–80.0)	75.7

## 4 Discussion

We developed a novel model for the estimation of mammographic sensitivity as a continuous function of tumor size, given that mammography’s detection capability varies according to tumor size. Therefore, such a model provides more details about the sensitivity of mammography screening. Aggregated data of 22,915 screen-detected and 10,670 interval cancers from the Dutch screening program were used to obtain the size distribution of detected as well as missed breast cancers at the time of screening. The estimated sensitivity showed an increase from 0 to 85% for tumors between 2 and 20 mm. A sensitivity analysis for the model indicated that TVDT was an influential factor for sensitivity, and the assumption that a tumor will be detected in a biennial screening program within 2 screening rounds after one false-negative result was deemed appropriate.

In our model, the follow-up time was used to determine how long the expected time was that allows all false-negatives to be detected, so that sensitivity was not overestimated due to underestimations of false negatives. In this study, the follow-up time was estimated based on the maximum delay time in diagnosis (1251 days) reported in Ciatto et al., and we used a follow-up time of 4 years which was rounded up from the value 1251 days [22]. Although this study dates from 1992 to 2001, we estimated that this data on follow-up times is still valid in the current state of screening programs. First, according to a recently published study which compared the median delay time between two time periods of 1997–2006 and 2007–2016 in the Netherlands [27], the median delay time for both periods was approximately 2 years which was similar to the reported median delay time in Ciatto et al. Importantly, the difference in delay time between the two periods was not statistically significant. Second, delayed diagnosis after false negative results is not only related to mammography sensitivity itself, but also related to participants compliance as shown in Ciatto et al. [22]. Third, the analysis on assumptions of our model showed that for a shorter follow-up time of 2 years, the screening sensitivity slightly increased, whereas for a longer follow-up time of 6 years, the sensitivity curve barely changed compared to that of 4 years. These results indicate that a follow-up time of 4 years is reasonable.

The validation of our model showed that the estimated sensitivity was comparable to two of the three studies [25,26], whereas the sensitivity was slightly underestimated compared to that of Cawson et al. [24]. A possible reason is that in the study of Cawson et al., only tumors that could be detected or were visible on mammograms were included. However, it is well-known that in a real-world screening setting a proportion of tumors is not detectable by mammography [18], which could explain the higher sensitivity reported. Although the model was generated based on data from Dutch breast cancer screening program, we anticipated that this model could also be applicable globally to other organized population screening programs with biennial mammography like Norway and Australia. This assumption was informed by several studies which suggest that screening interval plays a vital role in estimating mammography sensitivity [28]. Second, in addition to tumor size, mammography sensitivity can also be affected by participants’ characteristics such as mammographic density, and technical factors such as interpretive skills of radiologists [29]. We expect that these factors might not differ much between the Netherlands and the other two countries, and therefore could be used as reliable sources for our external validation.

Compared to other models where a seemingly optimistic sensitivity of 100% for tumor diameters of 15–20 mm was estimated [[10], [11], [12], [13]], our model provides a more reliable sensitivity of 85% for a tumor diameter of 20 mm. Studies have shown that on average 20% of the screen-detected cancers were larger than 20 mm, and data from Germany showed that approximately 8% of the incident tumors in their population screening program were even larger than 50 mm [[14], [15], [16]]. Although infrequent screening or fast-growing cancers could partly explain these larger tumor sizes, it is unlikely that the sensitivity would reach a perfect sensitivity at a size of 20 mm. In addition, several studies have shown that some cancers will not be visible on mammograms even at a very large size >70 mm [18,30,31], as factors like sites where visualization is difficult (close to the thorax wall), and especially high-density breast tissue will lead to not-detectable tumors on mammograms [18,32].

**Fig. 3:**
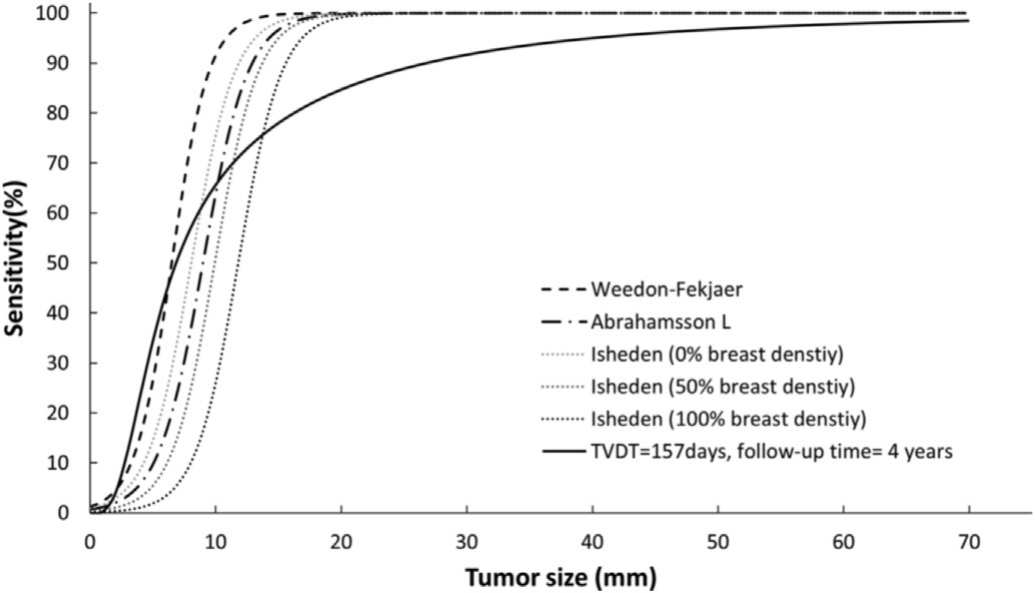
A comparison of the proposed sensitivity function model with other model studies. Data for 100%, 50%, and 0% breast density of Isheden et al. [12], of Weedon-Fakjaer et al. [10], and of Abrahamsson et al. [13]. TVDT = tumor volume doubling time.

The shape of the sensitivity curve in our study was similar to models estimated from logistic functions [Figs. 3, 10–13]. However, our model showed a higher sensitivity for tumors ≤10 mm, while the sensitivity became lower when the tumor size was larger than 10 mm. Possible reasons could be mainly explored from the model structure perspective. First, in our model, we did not make a prior assumption on the sensitivity function itself such as a logistic function that was used in other studies. By assuming a logistic function, the sensitivity would increase sharply at a certain point as observed in Fig. 3, which might lead to a higher sensitivity for larger tumors than that of our model. Second, certain aspects of the tumor growth model might be possible reasons. To be specific, in our model, we assumed that tumor grows through an exponential function with a constant TVDT, according to Collins et al. [19]. However, in Weedon-Fekjær et al., they used a growth model made by Spratt et al., in which tumor was assumed to grow through a logistic function with variation in individual growth rates [33,34]. In studies from Isheden et al. and Abrahamsson et al., although they also used an exponential model, the cell reproductive rate with a constant inverse growth rate was used as their parameters. Tumor growth rate had a crucial impact on the estimation of sensitivity as a faster growing tumor was more likely to be detected at a larger volume than a slower growing tumor, which might result in a higher sensitivity [35]. Nevertheless, it is difficult to compare in a straightforward way as conflicting results were reported on which model performs well and as different parameters were used to express tumor growth in these studies [36,37].

The differences between our model and the other models could be also explored from data perspective. Specifically, to estimate the tumor size distribution, we used relatively new data from 2004 to 2009, while Weedon-Fekjaer et al. estimated the parameters of the function by using screening data from 1995 to 2002 [10], and Isheden et al. and Abrahamsson et al. used same data from 1993 to 1995 [12,13]. As mammography detectability has been improved over time [38], and the modern mammography is able to detect more tumors at smaller size, a higher sensitivity at smaller size and also an overall lower sensitivity was observed in our study.

In addition to models that estimate screening sensitivity continuously as a function of tumor size, the MISCAN model calibrated mammographic sensitivity by T-stage. The sensitivity of our model was generally lower than the estimations of sensitivity in studies that used the MISCAN model [39,40]. For example, the estimated sensitivity at ≤5, 5–10, 10–20 and > 20 mm was 47%, 62%, 90% and 98% in Gelder et al. [39], while the estimated sensitivity for a tumor at 5, 10, and 20 mm in our model was 35%, 65%, and 85%. One of the possible reasons could be the inclusion of prevalent cancers detected at the first screening round [40], which might lead to a higher sensitivity in the MISCAN model.

The strengths of this study lie in several aspects. Firstly, unlike other modeling studies that assumed a logistic function [[10], [11], [12], [13]], we estimated a sensitivity model without any prior assumptions. Secondly, we used real-world aggregated data such as the number and size distribution of breast cancers, which can be relatively easily found in the national reports of breast cancer screening programs. Furthermore, the developed model can be easily adapted with different input parameters such as growth rates, different tumor size distributions and interval periods, which could make our sensitivity model useful for screening evaluation in other countries or other screening purposes.

However, there are also some limitations. Firstly, we used population-based data such as the number and size distribution of breast cancers based on T-stage, however, with a more detailed tumor size distribution instead of just the T-stage, the estimation of the lognormal distribution parameters would be more reliable. Secondly, the sensitivity model used a constant tumor diameter doubling time for every tumor, while in reality, the tumor growth varies widely among tumors or even for one tumor at different times [19,41]. Ideally, a comprehensive growth model could be incorporated if more detailed data were available. Thirdly, in our model, we assumed that all false-negative cancers would be detected in the future and became larger over time. However, studies have shown that some cancers will stop growing and even regress, which might lead to an overestimation of the sensitivity [42]. On the other hand, some fast-growing cancers would be recognized as false-negatives instead of new incident cancers, which might underestimate sensitivity [43]. Moreover, we assumed that the time between two screening rounds and the time between the last screening and an interval cancer was fixed. However, knowledge about these time distributions would enable us to better estimate the distribution of tumor sizes at time of screening. Lastly, in addition to tumor size, breast density and age also has an impact on mammographic sensitivity. Studies have shown that mammography sensitivity decreases in women with dense breasts and younger women [44]. The model presented here gives the sensitivity of mammography as used in screening settings for a population of women with mixed breast density and age. However, the sensitivity as a function of tumor size could in principle also be calculated for women with dense or fatty breasts or for different age groups if specific data on these groups of women were available [12].

## 5 Conclusion

In this study, we developed a model which estimates the sensitivity of mammography as a function of tumor size without any prior assumptions about the function itself. The sensitivity model showed a similar sensitivity curve shape compared with studies that were estimated from logistic function [[10], [11], [12], [13]], but the estimates in our model had a better representation of data observed in other screening programs. Furthermore, as tumor growth is an influential factor for the estimation of sensitivity, future studies that provide more detailed information on tumor progression, such as tumor doubling times, would help in further refining sensitivity estimates. In summary, our work provides knowledge on the tumor size-specific sensitivity of mammography. Our sensitivity model can be incorporated in cost-effectiveness models aiming to evaluate breast cancer screening programs. A tumor size-specific sensitivity might improve the performance of cost-effectiveness modeling compared with models that use only a single value for mammographic sensitivity.

## Ethical approval

This article does not contain any studies with human participants or animals performed by any of the authors.

## Funding

This research did not receive any specific grant from funding agencies in the public, commercial, or not-for-profit sectors.

## Declaration of competing interest

The authors declare that they have no conflict of interest.
