# Supplementary material for: Mammographic sensitivity as a function of tumor size: A novel estimation based on population-based screening data
Source: Breast. 2020 Dec 9;55:69–74. doi: 10.1016/j.breast.2020.12.003 (PMC7753195; doi:10.1016/j.breast.2020.12.003)
Supplement: Multimedia component 1 [file mmc1.docx]

***Search strategy***

("Breast Neoplasms"[Mesh] OR breast cancer*[tiab] OR breast tumo*[tiab] OR breast carcinom*[tiab] OR mammary cancer*[tiab] OR cancer of breast[tiab] OR cancer of the breast[tiab] OR breast malign*[tiab] OR breast neoplasm*[tiab])
AND
("Mammography"[Mesh] OR mammogra*[tiab])
AND
("Sensitivity and Specificity"[Mesh] OR sensitiv*[tiab] OR false negative[tiab])
AND
(size[tiab] OR tumour siz*[tiab] OR tumor siz*[tiab] OR tumour diamet*[tiab] OR tumor diamet*[tiab] OR tumor volum*[tiab] OR tumour volum*[tiab])
AND
("Mass Screening"[MeSH] OR screen*[tiab])

**Figure S1** Flowchart of literature search
